# Improved Secret Image Sharing Scheme in Embedding Capacity without Underflow and Overflow

**DOI:** 10.1155/2015/861546

**Published:** 2015-08-17

**Authors:** Liaojun Pang, Deyu Miao, Huixian Li, Qiong Wang

**Affiliations:** ^1^School of Life Science and Technology, Xidian University, Xi'an 710071, China; ^2^Department of Computer Science, Wayne State University, Detroit, MI 48202, USA; ^3^School of Computer Science and Engineering, Northwestern Polytechnical University, Xi'an 710072, China

## Abstract

Computational secret image sharing (CSIS) is an effective way to protect a secret image during its transmission and storage, and thus it has attracted lots of attentions since its appearance. Nowadays, it has become a hot topic for researchers to improve the embedding capacity and eliminate the underflow and overflow situations, which is embarrassing and difficult to deal with. The scheme, which has the highest embedding capacity among the existing schemes, has the underflow and overflow problems. Although the underflow and overflow situations have been well dealt with by different methods, the embedding capacities of these methods are reduced more or less. Motivated by these concerns, we propose a novel scheme, in which we take the differential coding, Huffman coding, and data converting to compress the secret image before embedding it to further improve the embedding capacity, and the pixel mapping matrix embedding method with a newly designed matrix is used to embed secret image data into the cover image to avoid the underflow and overflow situations. Experiment results show that our scheme can improve the embedding capacity further and eliminate the underflow and overflow situations at the same time.

## 1. Introduction

Secret sharing was put forward by Blakley [1] and Shamir [2] in 1979, respectively. Due to the extensive application of images in our daily life, in 1995, Naor and Shamir [3] introduced the concept of secret sharing into the image field and proposed the first secret image sharing scheme which is also known as visual secret sharing. After that, much attention was paid to the secret image sharing. It is such a technology, in which a secret image is transformed into multiple shares or stego images, so that the secret image can be recovered with a certain set of shares or stego images. In this case, we can use these shares to substitute the secret image during its transmission or storage and this can prevent the attackers from obtaining the secret image by monitoring communication channels or breaking into the storage devices. Therefore, it is an effective method to ensure the security of the secret image during its transmission or storage, especially in the fields of military, commercial, financial, and so forth [3].

How to maximize the embedding capacity is an important issue in the secret image sharing field. The embedding capacity refers to the maximum amount of secret data that can be hidden in given cover images without affecting imperceptibility. For a given cover image, the larger the embedding capacity of the scheme is, the more secret information can be embedded. Therefore, in order to embed a given secret image, the larger the embedding capacity of the scheme is, the smaller size of cover images is needed, which also results in that less time and space are demanded during transmission and storage [4–14]. The embedding capacity of most existing schemes [4–13] is not more than (*t* − 3)/3 of the size of the cover image, where *t* is the threshold value. In order to further improve the embedding capacity, in 2010, Lin and Chan [14] used a new technique to share the secret image and the embedding capacity of their scheme is (*t* − 1)/3 of the size of the cover image. According to literatures that we can find, Lin and Chan's result is still the best one by now. However, there is also a drawback in Lin and Chan's scheme. That is, Lin and Chan's scheme is faced with the underflow and overflow situations [14]; that is to say, the value of pixels of the stego image may exceed the grayscale boundary, which results in that the secret image and the cover image cannot be revealed lossless [14]. Recently, Guo et al. [10], Chang et al. [11], and Ulutas et al. [13] have used different methods to eliminate this problem. Although the underflow and overflow situations have been well dealt with, the embedding capacities of all these schemes [10, 11, 13] are lower than that of Lin and Chan's scheme. How to improve the embedding capacity and at the same time eliminate the underflow and overflow situations is one of hot issues in the secret image sharing field.

Motivated by these concerns, we proposed a new image sharing scheme to increase the embedding capacity and at the same time to eliminate the underflow and overflow situations. In the proposed scheme, the differential coding, Huffman coding, binary to hexadecimal data converting, and the pixel mapping matrix (PMM) embedding methods are used together to meet the design aim. In more detail, the differential coding is applied to eliminate spatial redundancies of original secret data, and the Huffman coding is used to compress secret data. Meanwhile, data converting is used to adapt this scheme to the power-of-two Galois Field GF(2^4^). Through the above processes, the obtained data are smaller than the original one, and therefore, the embedding capacity can be improved because the processed data substituted the original ones in the later sharing and embedding steps. At last, the PMM method is used to embed processed secret data into the cover image to avoid the underflow and overflow situations, where a new pixel mapping matrix is designed based on the improved Exploiting Modification Direction (EMD) method [15, 16] and Sudoku method [17–20]. Compared with the existing schemes, the proposed scheme has higher embedding capacity and has no underflow and overflow problems. At the same time, the computational complexity of the proposed scheme is lower and the visual quality of the proposed scheme is higher.

The rest of this paper is organized as follows.  Section 2 reviews the related works.  Section 3 illustrates the proposed scheme in detail. In Section 4, the performance analysis and experimental results are presented. Finally, Section 5 shows conclusions.

## 2. Related Works

Secret image sharing (SIS), an application of the secret sharing concept in the image field, is an effective way to protect a secret image during its transmission and storage. In 1995, Naor and Shamir [3] proposed the first SIS scheme which utilizes the human's visual system and their scheme is known as visual secret sharing (VSS) [3, 21–25]. In Naor and Shamir's scheme [3], the secret image was transformed into multiple transparencies and it can be recovered by stacking a certain set of transparencies. Since then, many SIS schemes have been proposed. Through the SIS method, secret image data can be protected during their transmission or storage. Since the visual quality of the VSS schemes was limited, in 2002, Thien and Lin [26] proposed the computational secret image sharing (CSIS) [4–14, 26–38] in which the secret image is recovered by mathematical computation, that is the Lagrange interpolation operation. Generally speaking, the CSIS schemes can be divided into two types. One is to transform the secret image into meaningless images that are known as shadows (or shares) [26–32]. Since shadows look like noise and easily attract attackers' attention, it is conscious to think of hiding the secret image data in meaningful images, called stego images [4–14, 33–38]. This is the second type of CSIS. There are many CSIS schemes with steganography which have been proposed to generate stego images. Steganography is such a technique which embeds secret image data into cover images to generate meaningful stego images. Specifically, the secret image is shared first and then embedded into cover images by using different embedding methods, through which stego images are generated. Compared with cover images, stego images are generated without visual perceptible changes, which can prevent an invader from being aware of the existence of the secret image. Since images are widely used in our daily life and the protection of the image is becoming increasingly critical, the SIS can be widely used in many fields such as military, commercial, and financial. For example, for the sake of security, contacts in e-commerce can be transformed into multiple stego images and they can be protected from the intruders' eyes in this way.

Since Naor and Shamir [3] proposed the first SIS scheme that is known as VSS by using Shamir's threshold secret sharing scheme [2], various techniques and methods have been studied to design novel VSS schemes [21–25]. At the same time, in the VSS field, how to share multiple secret images [22, 23] and how to prevent against cheating [25] are research focuses. After the first CSIS scheme [26] was proposed, much attention has been paid on CSIS. Former CSIS schemes [26–32] generated meaningless shadows to hide secret image data. And after 2004, most CSIS schemes [4–14, 33–38] generate meaningful stego images to camouflage secret image data. Recently, in the CSIS field, one important hot issue is to solve the problem resulting from the inhomogeneity of the important degree of stego images when reconstructing the secret images [28–31, 33, 34], and the answers can be divided into three types: hierarchical threshold CSIS [33, 34], weighted CSIS [28–30], and essential CSIS [31]. The second hot issue is to improve the visual quality of CSIS [4–8, 10, 12–14, 26], and most of the existing schemes [4–8, 10, 12–14, 26] work on how to improve the PSNR (Peak Signal to Noise Ratio) value of CSIS schemes, because PSNR is an important parameter to determine whether the visual quality is good or not. The higher the PSNR is, the better the visual quality is. Also, SSIM (structural similarity) [39] has been used to evaluate the visual quality of the proposed scheme in terms of the human visual system and similarity, recently. The third hot issue is to enhance the ability of authentication, which aims to help in enhancing the security of schemes [4, 6, 7, 9, 29, 35]. Decreasing the authentication bits with high authentication ability is a significant challenge [9]. The fourth issue is to share color images securely [29, 34–36], and recovering the distortion-free secret image and enhancing the security are two main problems. The fifth hot issue is to enhance the embedding capacity [32, 36–38]. To embed a given secret image, the larger the embedding capacity is, the smaller cover images are needed, which also results in that less time and space are needed during transmission and storage. Thus, CSIS schemes that have large capacity have considerable potential for many cases, for example, when the bandwidth is limited or systems have critical real-time requirements. These issues stimulate people to research the CSIS scheme constantly.

The embedding capacity has a significant influence on the performance of CSIS schemes, and it is an important index to determine the amount of secret image data which can be hidden in one cover image. Generally, large embedding capacity means that the cover image can share a large secret image and thus it can decrease the need for cover images when the size of the secret image is constant. Therefore, the embedding capacity is related to the size of cover images, and it is usually expressed by the percentage of the size of cover images [14]. In 2004, Lin and Tsai [4] proposed a CSIS scheme that was the first one to generate meaningful stego images and consider the embedding capacity, and in their scheme, Shamir's threshold scheme [2] was used to implement the sharing of the secret image. According to the (*t*, *n*)-threshold secret sharing scheme of Shamir, in which *t* denotes the threshold value and *n* denotes the number of sharing participants, there are *t* coefficients in the Lagrange interpolating polynomial. The pixels of the secret image were shared directly. And Lin and Tsai [4] took the value of one pixel of the secret image as one coefficient in the Lagrange interpolating polynomial each time in the sharing phase. It means that one pixel of the secret image was shared at once. The least significant bits (LSB) embedding method [40–42] was used to embed values of the Lagrange interpolating polynomial into the cover image in the embedding phase. It means that one pixel of the secret image was embedded into four pixels of the cover image at once. The embedding capacity was 1/4 of the size of the cover image. However, the embedding capacity was not high [4] and the secret image cannot be retrieved without loss [8].

Similar to Lin and Tsai's scheme [4], recently, many CSIS schemes have been proposed, in which Shamir's scheme [2] was still used to share the secret image, but various methods were applied to the secret image before sharing and different embedding techniques were employed to generate stego images. In 2004, Wu et al. [5] proposed a new CSIS scheme to improve the embedding capacity. The scheme was the first one to apply the quantization procedure to process the secret image before the sharing phase, which can narrow the range of gray values of the secret image. In the embedding phase, the quantization method was used to embed values of the Lagrange interpolating polynomial into the cover image. The embedding capacity improved slightly and it was just bigger than 1/4 of the size of the cover image and the secret image cannot be retrieved without loss either [8]. Yang et al. [6] in 2007 and Chang et al. [7] in 2008 also used the LSB method to generate stego images, and the embedding capacities were still not large. In 2009, Lin et al. [8] proposed a new CSIS scheme with the modulo operation [43, 44] embedding. Before sharing, secret image pixels and cover image pixels were transformed into the 7-ary notational system to generate shared secret data and information data, respectively. Among them, shared secret data were shared in place of the secret image and information data were used to reconstruct cover images in the retrieving phase. In the sharing phase, (*t* − 3) transformed secret data were used as (*t* − 3) coefficients and 3 types of information data were used as the other 3 coefficients in the Lagrange interpolating polynomial each time. In the embedding phase, Lin et al. [8] utilized the modulus operator to embed values of the Lagrange interpolating polynomial into the cover image, which means (*t* − 3)/3 pixels of the secret image were embedded into one pixel of the cover image at once. It should be noted that the threshold value *t* is bigger than 3 in their scheme. The embedding capacity was (*t* − 3)/3 of the size of cover images, so it was improved. However, the embedding capacity was still limited and thus new technologies should be used to get high embedding capacity.

In 2010, Lin and Chan [14] proposed another new CSIS scheme with quantification and modulo operation. Compared with their early scheme [8], information data were generated by modulo operation. Each cover pixel was transformed into one information datum, which can decrease the number of information data. Compared with their early scheme [8], in the sharing phase, the number of coefficients that denoted information data is decreased from 3 to 1, while the number of coefficients that denoted shared secret data is increased from (*t* − 3) to (*t* − 1). It means that more shared secret data were shared at once. In embedding phase, Lin and Chan [14] also used quantification to embed values of the Lagrange interpolating polynomial into the cover image. Thus, more information of the secret image was embedded into one pixel of the cover image, which can improve the embedding capacity largely. Lin and Chan [14] noted that setting the prime number to 7 can achieve a balance between capacity and distortion. Thus, the prime number is set to 7 and the embedding capacity is (*t* − 1)/3 of the size of the cover image. However, Lin and Chan [14] also pointed out that their scheme had the underflow and overflow situations. Because the shared secret data were camouflaged into quantized cover image data, the value of pixels of the stego image may exceed the grayscale boundary. Thus, the secret image cannot be revealed without distortion. Later, many other CSIS schemes that used new technologies have been proposed, but the embedding capacities were all lower than that of Lin and Chan's scheme [14].

As mentioned above, the underflow and overflow situations can lead to the distortion of the recovered secret image, so how to prevent this problem should be considered when improving the embedding capacity. Using other embedding technologies, some CSIS schemes have been proposed without the problem. Pixel mapping matrix (PMM) embedding [11] is such a technology that cannot cause the underflow and overflow situations. The PMM is a 2-dimensional hypercube, which is 256 × 256. In the matrix, coordinate figures of the *x*-axis and *y*-axis represent the pixel value of the gray scale ranged from 0 to 255. The values in the pixel mapping matrix are determined by the algorithm that constructs the pixel mapping matrix. According to the designed arithmetic, every two pixels can be mapped to one value in the pixel mapping matrix and each value in the pixel mapping matrix can be mapped to a pair of pixels, so the PMM embedding method can prevent the underflow and overflow situations.

In 2010, Chang et al. [11] proposed a CISIS scheme with PMM embedding firstly. In Chang et al.'s scheme, PMM was designed by a Sudoku grid [17–20]. Sudoku is a logic-based number placement puzzle which is shown on a square grid. The PMM was divided into 4 × 4 blocks. Every block consists of different numbers ranging from 0 to 15. Chang et al. [11] transformed the secret image pixels into the 2^4^-ary notational system, while information data were generated according to the PMM because it can preserve the feature of the cover pixel pair. But their scheme has no good compression in the prepossessing phase; the embedding capacity was only (*t* − 1)/4 of the size of the cover image. The secret image and cover image can be distortion-free revealed. But the pixel mapping matrix is designed with high computational complexity. In 2011, Guo et al. [10] proposed a new CSIS scheme that also used the PMM embedding method. Guo et al. [10] introduced the exploiting modification direction (EMD) method [15, 16] to CSIS and utilized the EMD method to design the PMM, which was divided into some 1 × 5 or 5 × 1 blocks. Every block was composed of different numbers ranged from 0 to 4. Guo et al. [10] transformed the secret image pixels into the 5-ary notational system and information data were generated according to the PMM. However, Guo et al. [10] pointed that the dividing method resulted in that not all pixels in the cover image can be used to camouflage secret data. The embedding capacity was smaller than (*t* − 1)/4 of the size of the cover image. In 2013, Ulutas et al. [13] used a modified EMD to embed secret image into the cover image, which also can prevent the underflow and overflow situations. Because the range of stego image pixels changed largely, the embedding capacity was only 1/4 of the size of the cover image. That is, though the underflow and overflow situations can be prevented from above schemes, their embedding capacities were all lower than Lin and Chan's scheme [14].

By the above analysis, the existing scheme, which has the highest embedding capacity, has the underflow and overflow situations. While some schemes can prevent the underflow and overflow situations, their embedding capacity dropped dramatically. Motivated by these concerns, the proposed scheme aims to improve the embedding capacity further and prevent the underflow and overflow situations at the same time.

## 3. The Proposed Scheme

To implement high embedding capacity and eliminate the underflow and overflow situations at the same time, based on the inspiration of the previous schemes and the analysis of their pros and cons, we propose a new (*t*, *n*)-threshold secret image sharing scheme. In the proposed scheme, the differencing function, Huffman coding, and data converting are applied to compress the secret image firstly, and then the PMM embedding method with a newly designed pixel mapping matrix is used to embed compressed secret image data into the cover image. In order to facilitate describing the proposed scheme, we shall introduce notations used in the whole paper firstly, and they are shown in Notations.

**Figure 1:**
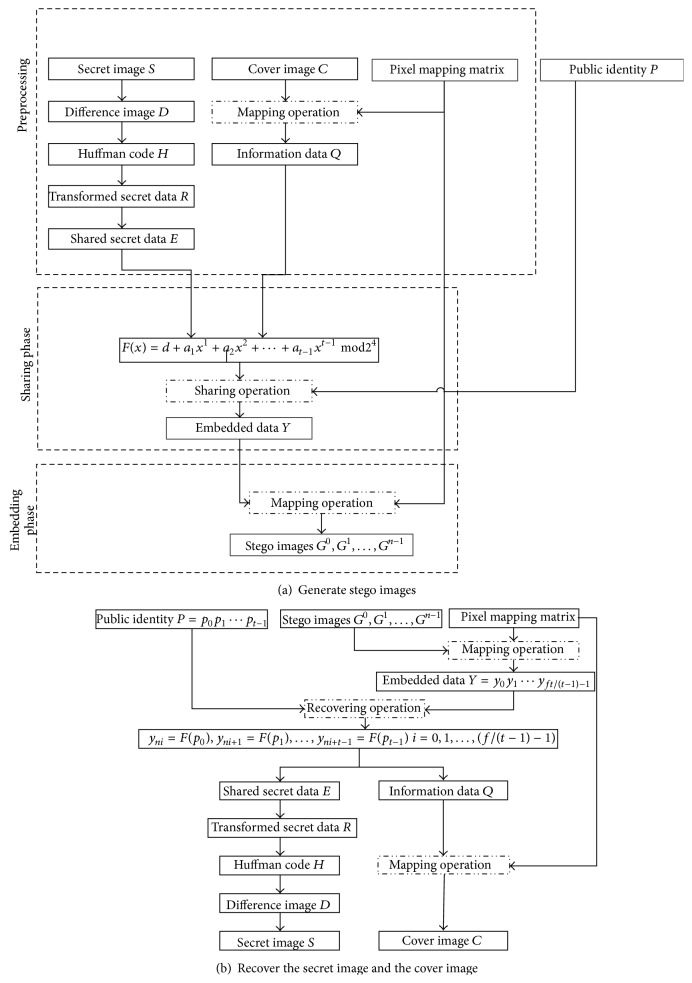
The flowchart of the proposed scheme.

The proposed scheme consists of four parts that are* the preprocessing phase*,* the sharing phase*,* the embedding phase*, and* the retrieving phase*, which can be demonstrated by Figure 1 and explained in Sections 3.1–3.4 in more detail, respectively.

In the proposed scheme, there are *n* participants *p*_0_, *p*_1_,…, *p*_*n*−1_ and without loss of generality, we can set the public identity of the *i*th participant *p*_*i*−1_ to be *i* for simplicity. That is to say, public identity *P* = {*p*_0_, *p*_1_,…, *p*_*n*−1_} = {1, 2,…, *n*}, in which *p*_*i*_ ∈ {1, 2,…, *n*} and *p*_*i*−1_ = *i*. Without loss of generality, in the recovery phase, we select the first *t* participants with the first *t* stego images *G* ^0^, *G* ^1^,…, *G* ^*t*−1^ and the first *t* participants *p*_0_ = 1, *p*_1_ = 2,…, *p*_*t*−1_ = *t* to recover the secret image and the cover image.

### 3.1. The Prepossessing Phase

In the preprocessing phase, we should process three things. One is to compress the secret image to generate the shared secret data *E*. The shared secret data *E* is used in the sharing process instead of the secret image *S*. The second is to design the pixel mapping matrix, which is used to generate information data *Q* = *q*_0_ *q*_1_ ⋯ *q*_*m*−1_, *q*_*i*_ ∈ {0,1,…, 15}. Note: the matrix also acts as the embedding rules later. The last is to generate information data *Q* based on the above matrix, and the information data *Q* will act as the helper data for the data sharing and retrieving.

#### 3.1.1. The Compression Process

By using the differencing function, the Huffman coding, and the data converting, the secret image *S* = {*s*_*i*,*j*_} is transformed into the shared secret data *E* = *e*_0_ *e*_1_ ⋯ *e*_*f*−1_, which is shown as follows.

**Step 1 .**

The differencing function [27] is applied to the secret image *S* to get the difference image *D* = {*d*_*i*,*j*_∣*i* = 0,1,…, *M*_*S*_ − 1,  *j* = 0,1,…, *N*_*S*_ − 1,  *d*_*i*,*j*_ ∈ {−255, −254,…, 255}}, which helps to eliminate spatial redundancies and ensure the good quality of compression. Set the difference vector *B* = (*b*_0_, *b*_1_,…, *b*_*M*_*S*_×*N*_*S*_−1_) = (*d*_0,0_, *d*_0,1_,…, *d*_0,*N*_*S*_−1_, *d*_1,0_, *d*_1,1_,…, *d*_1,*N*_*S*_−1_,…, *d*_*M*_*S*_−1,0_, *d*_*M*_*S*_−1,1_,…, *d*_*M*_*S*_−1,*N*_*S*_−1_).

**Step 2 .**

The Huffman coding [27] is put on *B* to get Huffman code *H* = *h*_0_ *h*_1_ ⋯ *h*_len−1_ with the bit length len, *h*_*i*_ ∈ {0,1}. In order to reveal the secret image *S* = {*s*_*i*,*j*_} later, the probability of each element in the difference vector *B* = *b*_0_ *b*_1_ ⋯ *b*_*M*_*S*_×*N*_*S*_−1_ should be recorded for retrieving the secret image later.

**Step 3 .**

A data converting is put on Huffman code *H* to get the transformed secret data *R* = *r*_0_ *r*_1_ ⋯ *r*_*u*−1_, *r*_*i*_ ∈ {0,1,…, 15}, where *u* = ⌈len/4⌉, which can adapt this scheme to the power-of-two Galois Field GF(2^4^) well. In our scheme, firstly, dividing Huffman codes *H* = *h*_0_ *h*_1_ ⋯ *h*_len−1_ into nonoverlapping segments 4 bits by 4 bits and *u* = ⌈len/4⌉ segments are obtained. And then, set *r*_0_ = BTH(*h*_0_ *h*_1_ *h*_2_ *h*_3_), *r*_1_ = BTH(*h*_4_ *h*_5_ *h*_6_ *h*_7_),…, *r*_*u*−1_ = BTH(*h*_4(*u* − 1)_ ⋯ *h*_len−1_).

**Step 4 .**

The shared secret data *E* = *e*_0_ *e*_1_ ⋯ *e*_*f*−1_, *f* = *u* + (*t* − 1 − (*u* + 6)%(*t* − 1)) + 6, which can be obtained as follows: the first *u* values in shared secret data *E* are from the transformed secret data *R* = *r*_0_ *r*_1_ ⋯ *r*_*u*−1_. That is to say, *e*_*i*_ is equal to *r*_*i*_ in value for *i* = 0,1,…, *u* − 1; and the last 6 values are used to record the value of len in order to help to recover the secret data without any loss; other values are set to be 0. Such that *f* is an integer multiple of (*t* − 1).

By now, the shared secret data *E* = *e*_0_ *e*_1_ ⋯ *e*_*f*−1_ is got.

#### 3.1.2. To Create the Pixel Mapping Matrix

The pixel mapping matrix is used to generate information data *Q* and also acts as the embedding rules later. The pixel mapping matrix is a 2-dimensional hypercube, which is 256 × 256 and coordinate figures of the *k*-axis and *l*-axis represent the pixel value of the gray scale ranged from 0 to 255, respectively. In the proposed scheme, the element mat_*k*,*l*_ in the pixel mapping matrix is defined by

(1) matk,l=l+4×kmod⁡24,where  k,l=0,1,…,255.

**Figure 2:**
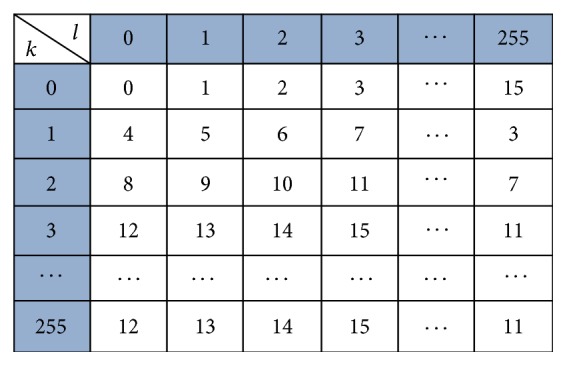
The proposed matrix.

By the above method, the proposed pixel mapping matrix can be shown as Figure 2.

The proposed pixel mapping matrix has two advantages: it can be divided into 4096 4 × 4 blocks, which can use all pixels of the cover image to camouflage secret data in the embedding phase; in each 4 × 4 block, the difference values of coordinate figures of the *k*-axis and *l*-axis are all in the range of [−3,3], which is small and can help to increase the visual quality.

#### 3.1.3. The Generation of Information Data *Q*

Information data *Q*, which acts as the helper data for the data sharing and retrieving, is generated from the cover image *C* = {*c*_*i*,*j*_∣*i* = 0,1,…, *M*_*C*_ − 1,  *j* = 0,1,…, *N*_*C*_ − 1,  *c*_*i*,*j*_ ∈ {0,1,…, 255}} by using the designed pixel mapping matrix in Section 3.1.2. From the cover image *C*, we set the cover vector *W* = (*w*_0_, *w*_1_,…, *w*_*M*_*C*_×*N*_*C*_−1_) = (*c*_0,0_, *c*_0,1_,…, *c*_0,*N*_*C*_−1_, *c*_1,0_, *c*_1,1_,…, *c*_1,*N*_*C*_−1_,…, *c*_*M*_*C*_−1,0_, *c*_*M*_*C*_−1,1_,…, *c*_*M*_*C*_−1,*N*_*C*_−1_). To obtain the information data *Q* = *q*_0_ *q*_1_ ⋯ *q*_*m*−1_, where *m* = *f*/(*t* − 1), the following steps should be repeated for *i* = 0,1,…, *m* − 1.

**Step 1 .**

Get the pixel pair (*w*_2*i*_, *w*_2*i*+1_) in the cover vector *W*.

**Step 2 .**

**Figure 3:**
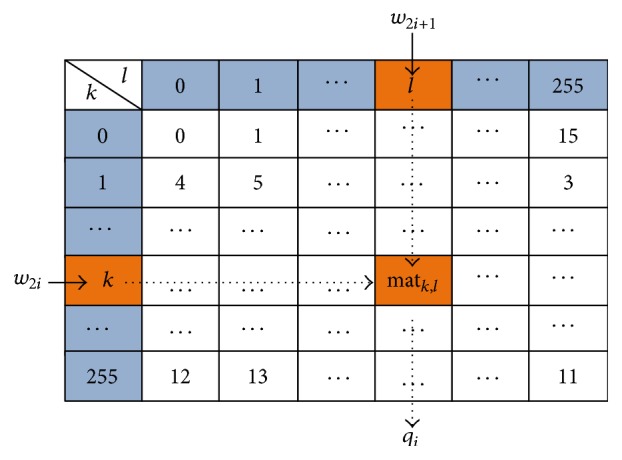
To obtain the information data *Q*.

Set *k* = *w*_2*i*_ and *l* = *w*_2*i*+1_, and get the value mat_*k*,*l*_ in the above matrix. Then, set *q*_*i*_ = mat_*k*,*l*_, which can be shown in Figure 3.

By now, the information data *Q* = *q*_0_ *q*_1_ ⋯ *q*_*m*−1_ can be got.

### 3.2. Sharing Phase

In the sharing phase, we shall transform the shared secret data *E* and the information data *Q* into the embedded data *Y* = *y*_0_ *y*_1_ ⋯ *y*_*v*−1_, *v* = *f* × *n*/(*t* − 1), *y*_*i*_ ∈ {0,1,…, 15} by using Shamir's threshold scheme [2], and the embedded data *Y* will be embedded in the cover image *C* later.

To obtain the embedded data *Y* = *y*_0_ *y*_1_ ⋯ *y*_*v*−1_, the following steps should be repeated for *i* = 0,1,…, (*f*/(*t* − 1) − 1).

**Step 1 .**

Get the (*t* − 1) data *e*_(*t* − 1)*i*_, *e*_(*t* − 1)*i*+1_,…, *e*_(*t*−1)*i*+*t*−2_ in shared secret data *E* = *e*_0_, *e*_1_,…, *e*_*f*−1_, and let *a*_*j*_ = *e*_(*t*−1)*i*+*j*−1_ for *j* = 1,2,…, *t* − 1. Get the value *q*_*i*_ in the information data *Q* = *q*_0_ *q*_1_ ⋯ *q*_*m*−1_ and set *d* = *q*_*i*_. Then, construct the following:

(2) $$\begin{array}{c} F\left(\;x\;\right)=d+a_{1}x+a_{2}x^{2}+\cdots +a_{t-1}x^{t-1}\mathrm{mod}2^{4}. \end{array}$$

**Step 2 .**

Compute the following:

(3) $$\begin{array}{c} y_{ni}=F\left(\;p_{0}\;\right)=F\left(\;1\;\right),\\ y_{ni+1}=F\left(\;p_{1}\;\right)=F\left(\;2\;\right),\\ \vdots \\ y_{ni+n-1}=F\left(\;p_{n-1}\;\right)=F\left(\;n\;\right). \end{array}$$

By now, all the embedded data *Y* = *y*_0_ *y*_1_ ⋯ *y*_*v*−1_ can be got.

### 3.3. Embedding Phase

In the embedding phase, the embedded data *Y* are embedded into the cover image *C* to generate *n* stego images *G* ^0^, *G* ^1^,…, *G* ^*n*−1^, where stego image *G* ^*i*^ is the hold by the *i*th participant *p*_*i*−1_. To simplify description, we denote stego image *G* ^*i*^ as *G* ^*i*^ = {*g*_*k*,*l*_^*i*^∣*k* = 0,1,…, *M*_*C*_ − 1,  *l* = 0,1,…, *N*_*C*_ − 1,  *g*_*k*,*l*_^*i*^ ∈ {0,1,…, 255}}. Set the stego vector *Z* ^*i*^ = (*z*_0_^*i*^, *z*_1_^*i*^,…, *z*_*M*_*C*_×*N*_*C*_−1_^*i*^) = (*g*_0,0_^*i*^, *g*_0,1_^*i*^,…, *g*_0,*N*_*C*_−1_^*i*^, *g*_1,0_^*i*^, *g*_1,1_^*i*^,…, *g*_1,*N*_*C*_−1_^*i*^,…, *g*_1,*M*_*C*_−1,0_^*i*^, *g*_1,*M*_*C*_−1,1_^*i*^,…, *g*_*M*_*C*_−1,*N*_*C*_−1_^*i*^).

To obtain the *n* stego images *G* ^0^, *G* ^1^,…, *G* ^*n*−1^, the process is shown as follows.

**Step 1 .**

To get the first 2*f*/(*t* − 1) values of each stego vector *Z* ^0^, *Z* ^1^,…, *Z* ^*n*−1^, respectively, the following steps should be repeated for *i* = 0,1,…, (*f*/(*t* − 1) − 1):

(1) Get the *i*th pixel pair in the cover vector *W* = *w*_0_ *w*_1_ ⋯ *w*_*M*_*C*_×*N*_*C*_−1_, denoted by (*w*_2*i*_, *w*_2*i*+1_) and the corresponding information datum *q*_*i*_ in the information data *Q*, and then we can determine a 4 × 4 block which contains 16 different values.
(2) Get *n* data *y*_*ni*+*j*_, for *j* = 0,1,…, *n* − 1, from the embedded data *Y*, and locate each of these data *y*_*ni*_, *y*_*ni*+1_,…, *y*_*ni*+*n*−1_ in the 4 × 4 block, respectively; the *i*-axis of the value *y*_*ni*+*j*_ is the value of *z*_2*i*_^*j*^, and the *j*-axis of the value *y*_*ni*+*j*_ is the value of *z*_2*i*+1_^*j*^.

Thus, the first 2*f*/(*t* − 1) values of each stego vector *Z* ^0^, *Z* ^1^,…, *Z* ^*n*−1^ can be got, respectively.

**Step 2 .**

To get the other values of each stego vector *Z* ^0^, *Z* ^1^,…, *Z* ^*n*−1^, the following step should be repeated for *i* = 0,1,…, *n* − 1:

(1) Let *z*_*j*_^*i*^ = *w*_*j*_, for *j* = 2*f*/(*t* − 1), 2*f*/(*t* − 1) + 1,…, *M*_*C*_ × *N*_*C*_ − 1.

Thus, the other values of each stego vector *Z* ^0^, *Z* ^1^,…, *Z* ^*n*−1^ can be got.

**Step 3 .**

To get *n* stego images *G* ^0^, *G* ^1^,…, *G* ^*n*−1^, set (*g*_0,0_^*i*^, *g*_0,1_^*i*^,…, *g*_0,*N*_*C*_−1_^*i*^, *g*_1,0_^*i*^, *g*_1,1_^*i*^,…, *g*_1,*N*_*C*_−1_^*i*^,…, *g*_*M*_*C*_−1,0_^*i*^, *g*_*M*_*C*_−1,1_^*i*^,…, *g*_*M*_*C*_−1,*N*_*C*_−1_^*i*^) = (*z*_0_^*i*^, *z*_1_^*i*^  ,…, *z*_*M*_*C*_,*N*_*C*_−1_^*i*^) for *i* = 0,1,…, *n* − 1.

Thus, we can get all stego images *G* ^0^, *G* ^1^,…, *G* ^*n*−1^, which should be given to *n* participants, respectively.

Also, we take an example of generating stego images. The example not only can show the process of how to generate stego images, but also can show that the problem of underflow and overflow situations does not occur in the proposed scheme.

Suppose that we use a (2, 4)-threshold secret image sharing scheme, and we assume the first data of shared secret data *E* is 6, and the first cover pixel pair is (255, 255). The inputs are a shared secret datum 6 and one cover pixel pair (255, 255) and the outputs are 4 stego pixel pairs. The detail process is shown as follows.

**Step 1 .**

The cover pixel pair (255, 255) is mapped into the information datum 11 according to the pixel mapping matrix.

**Step 2 .**

In the sharing phase, we shall take the first shared secret data *E* = 6 and the information data *Q* = 12 into the Lagrange interpolation polynomial, as the following formula:

(4) $$\begin{array}{c} F\left(\;x\;\right)=11+6x\mathrm{mod}2^{4}. \end{array}$$

**Step 3 .**

Assume the public identity of 4 participants are *p*_1_ = 1, *p*_2_ = 2, *p*_3_ = 3, and *p*_4_ = 4, and all the embedded data can be got. The values of the embedding data are *y*_1_ = 1, *y*_2_ = 7, *y*_3_ = 13, and *y*_4_ = 3, respectively.

**Step 4 .**

For the first pixel pair (255, 255) in the cover image, the unique 4 × 4 block in pixel mapping matrix is determined, which contains 16 different values in the pixel mapping matrix. It is shown in Table 1.

**Step 5 .**

We take each of these data *y*_1_ = 1, *y*_2_ = 7, *y*_3_ = 13, and *y*_4_ = 3 into the 4 × 4 block, respectively; then we can obtain four pixel pairs (253, 253), (254, 255), (252, 253), and (253, 255), respectively, and it is shown in Table 2.

Thus, the first two pixels of these four stego images are (253, 253), (254, 255), (252, 253), and (253, 255), respectively.

### 3.4. Retrieving Phase

In the retrieving phase, all pixels in the secret image and cover image could be recovered completely by using any *t* or more than *t* stego images. Without loss of generality, the first *t* stego images *G* ^0^, *G* ^1^,…, *G* ^*t*−1^ are selected. The processes of recovering the secret image *S* = {*s*_*i*,*j*_∣*i* = 0,1,…, *M*_*S*_ − 1,  *j* = 0,1,…, *N*_*S*_ − 1,  *s*_*i*,*j*_ ∈ {0,1,…, 255}} and the cover image *C* = {*c*_*i*,*j*_∣*i* = 0,1,…, *M*_*C*_ − 1,  *j* = 0,1,…, *N*_*C*_ − 1,  *c*_*i*,*j*_ ∈ {0,1,…, 255}} are shown in Sections 3.4.1 and 3.4.2, respectively.

#### 3.4.1. To Recover the Secret Image

By using stego images *G* ^0^, *G* ^1^,…, *G* ^*t*−1^, the secret image *S* = {*s*_*i*,*j*_} is recovered as follows.

**Step 1 .**

Let *Z* ^*i*^ = (*z*_0_^*i*^, *z*_1_^*i*^,…, *z*_*M*_*C*_×*N*_*C*_−1_^*i*^) = (*g*_0,0_^*i*^, *g*_0,1_^*i*^,…, *g*_0,*N*_*C*_−1_^*i*^, *g*_1,0_^*i*^, *g*_1,1_^*i*^,…, *g*_1,*N*_*C*_−1_^*i*^,…, *g*_*M*_*C*_−1,0_^*i*^, *g*_*M*_*C*_−1,1_^*i*^,…, *g*_*M*_*C*_−1,*N*_*C*_−1_^*i*^), for *i* = 0,1,…, *t* − 1. Thus, *t* stego vectors *Z* ^0^, *Z* ^1^,…, *Z* ^*t*−1^can be got.

**Step 2 .**

To get the embedded data *Y* = *y*_0_ *y*_1_ ⋯ *y*_*ft*/(*t*−1)−1_, the following step should be repeated for *i* = 0,1,…, (*f*/(*t* − 1) − 1):

(1) Get the *i*th pixel pair in the *j*th vectors *Z* ^*j*^, denoted as (*z*_2*i*_^*j*^, *z*_2*i*+1_^*j*^), and set *k* = *z*_2*i*_^*j*^ and *l* = *z*_2*i*+1_^*j*^. Then, set *y*_*ti*+*j*_ equal to mat_*k*,*l*_ in value, for *j* = 0,1,…, *t* − 1.

Thus, the embedded data *Y* = *y*_0_ *y*_1_ ⋯ *y*_*ft*/(*t*−1)−1_ can be got.

**Step 3 .**

To get the shared secret data *E* = *e*_0_ *e*_1_ ⋯ *e*_*f*−1_, the following steps should be repeated for *i* = 0,1,…, (*f*/(*t* − 1) − 1):

(1) Compute the following:
  (5) yti=Fp0=F1,yti+1=Fp1=F2,⋮yti+t−1=Fpt−1=FtFpi=Fi+1=d+a1i+1+a2i+12+⋯+at−1i+1t−1mod⁡24.
(2) Let *e*_(*t*−1)*i*+*j*−1_ = *a*_*j*_, and *q*_*i*_ = *d*, for *j* = 1,2,…, *t* − 1.

Thus, the shared secret data *E* = *e*_0_ *e*_1_ ⋯ *e*_*f*−1_ and the information data *Q* = *q*_0_ *q*_1_ ⋯ *q*_*m*−1_ can be got.

**Step 4 .**

According to the last 6 values in the shared secret data *E*, the value of len can be got.

**Step 5 .**

Set *u* = ⌈len/4⌉, and the transformed secret data *R* = *r*_0_ *r*_1_ ⋯ *r*_*u*−1_ can be got from the first *u* values in shared secret data *E*. That is to say, *r*_*i*_ is equal to *e*_*i*_ in value for *i* = 0,1,…, *u* − 1.

**Step 6 .**

Huffman code *H* = *h*_0_ *h*_1_ ⋯ *h*_len−1_ can be got from transformed secret data *R* = *r*_0_ *r*_1_ ⋯ *r*_*u*−1_. Set (*h*_0_ *h*_1_ *h*_2_ *h*_3_) = HTB(*r*_0_), (*h*_4_ *h*_5_ *h*_6_ *h*_7_) = HTB(*r*_1_),…, (*h*_4(*u* − 1)_ ⋯ *h*_len−1_) = HTB(*r*_*u*−1_).

**Step 7 .**

Decode the Huffman code *H* to reveal difference vector *B* = *b*_0_ *b*_1_ ⋯ *b*_*M*_*S*_×*N*_*S*_−1_, and get the difference image *D* = (*d*_0,0_, *d*_0,1_,…, *d*_0,*N*_*S*_−1_, *d*_1,0_, *d*_1,1_,…, *d*_1,*N*_*S*_−1_,…, *d*_*M*_*S*_−1,0_, *d*_*M*_*S*_−1,1_,…, *d*_*M*_*S*_−1,*N*_*S*_−1_) = (*b*_0_, *b*_1_,…, *b*_*M*_*S*_×*N*_*S*_−1_).

**Step 8 .**

The reverse differencing function [27] is applied to the difference image *D* = {*d*_*i*,*j*_} to get the secret image *S* = {*s*_*i*,*j*_}.

Thus, the secret image *S* = {*s*_*i*,*j*_} can be got.

#### 3.4.2. To Recover the Lossless Cover Image

By using stego images *G* ^0^, *G* ^1^,…, *G* ^*t*−1^, the cover image *C* = {*c*_*i*,*j*_∣*i* = 0,1,…, *M*_*C*_ − 1, *j* = 0,1,…, *N*_*C*_ − 1,  *c*_*i*,*j*_ ∈ {0,1,…, 255}} can be recovered. The information data *Q* = *q*_0_ *q*_1_ ⋯ *q*_*m*−1_ can be got in Section 3.4.1. In order to obtain the cover image *C* = {*c*_*i*,*j*_}, the process is as follows.

**Step 1 .**

**Figure 4:**
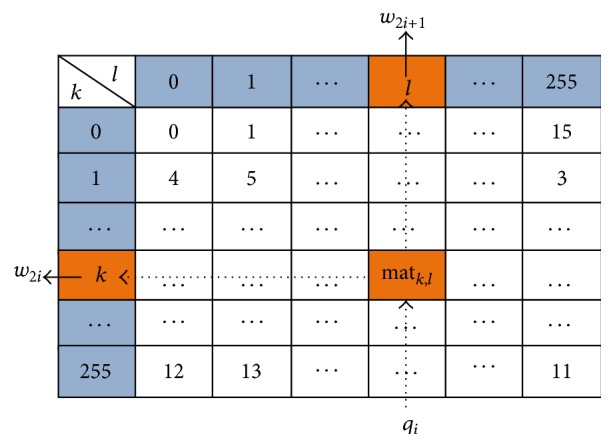
To obtain the cover vector *W*.

To get the first 2*m* values in the cover vector *W* = *w*_0_ *w*_1_ ⋯ *w*_*M*_*C*_×*N*_*C*_−1_, the following steps should be repeated for *i* = 0,1,…, *m* − 1:

(1) Get the *i*th pixel pair in the vector *Z* ^0^, denoted as (*z*_2*i*_^0^, *z*_2*i*+1_^0^), and *q*_*i*_ in the information data *Q* = *q*_0_ *q*_1_ ⋯ *q*_*m*−1_. Then, the unique 4 × 4 block with 16 various numbers in the pixel mapping matrix can be determined.
(2) Set mat_*k*,*l*_ = *q*_*i*_, where *w*_2*i*_ is equal to the value of *k*, and *w*_2*i*+1_ is equal to the value of *l*, which can be shown in Figure 4.

The first 2*m* values in the cover vector *W* = *w*_0_ *w*_1_ ⋯ *w*_*M*_*C*_×*N*_*C*_−1_ can be got.

**Step 2 .**

The other values: the last (*M*_*C*_ × *N*_*C*_ − 2*m*) values in the cover vector *W* = *w*_0_ *w*_1_ ⋯ *w*_*M*_*C*_×*N*_*C*_−1_ are the same as last (*M*_*C*_ × *N*_*C*_ − 2*m*) values in the vector *Z* ^0^. That is to say, *w*_*i*_ is equal to *z*_*i*_^0^ in values for *i* = 2*m*, 2*m* + 1,…, *M*_*C*_ × *N*_*C*_ − 1.

**Step 3 .**

Get the cover image *C* = (*c*_0,0_, *c*_0,1_,…, *c*_0,*N*_*C*_−1_, *c*_1,0_, *c*_1,1_,…, *c*_1,*N*_*C*_−1_,…, *c*_*M*_*C*_−1,0_, *c*_*M*_*C*_−1,1_,…, *c*_*M*_*C*_−1,*N*_*C*_−1_) = (*w*_0_, *w*_1_,…, *w*_*M*_*C*_×*N*_*C*_−1_).

Thus, the cover image *C* = {*c*_*i*,*j*_} can be got.

## 4. Performance Analysis and Experimental Results 

In this section we firstly theoretically analyze the performance of the proposed scheme and compare with Lin and Chan's scheme [14] which has the largest embedding capacity in the existing schemes, including the embedding capacity and the visual quality in Section 4.1. And then five experiments have been done to show the good performance of the proposed scheme from above two aspects in Section 4.2.

### 4.1. Performance Analysis

The embedding capacity is related to the needed time and demanded space during transmission and storage [14], and the visual quality is related to the security of the scheme, and it is an essential standard in CSIS schemes. The performance analysis of the proposed scheme and its comparison with Lin and Chan's scheme [14] is shown in the following two aspects.

#### 4.1.1. Embedding Capacity

The embedding capacity EC can be defined as follows:

(6) $$\begin{array}{c} \text{EC}=M_{C}\times N_{C}\times J\times \text{RC}. \end{array}$$

*J* is usually determined by the concrete embedding method. RC is the ratio of compression, which aims to show the compression effect, and is defined as follows:

(7) $$\begin{array}{c} \text{RC}=\frac{M_{S}\times N_{S}}{f}, \end{array}$$

where the value of *f* is the number of elements in the shared secret data *E*. Since the existence of the compression process, the shared secret data *E* = *e*_0_ *e*_1_ ⋯ *e*_*f*−1_ instead of the secret image *S* = {*s*_*i*,*j*_} are shared by the Lagrange interpolating polynomial. The number of the shared secret data *E* = *e*_0_ *e*_1_ ⋯ *e*_*f*−1_, say *f*, is smaller than the number of data of the secret image *S* = {*s*_*i*,*j*_}; thus the number of the embedded data *Y* = *y*_0_ *y*_1_ ⋯ *y*_*v*−1_ can be decreased dramatically, which results in that the larger secret image can be embedded into the determined cover image. Therefore, the embedding capacity can be improved accordingly. The smaller the value of *f* is, the larger the ratio of compression RC is, which can result in the higher embedding capacity EC.

In the following, we shall evaluate the embedding capacity of Lin and Chan's scheme and ours by the above evaluative criteria.

In Lin and Chan's scheme [14], the pixels of the secret image are transformed into the 7-ary notational system before sharing. And (*t* − 1) shared secret data can be embedded into one pixel of the cover image. We evaluate its embedding capacity EC as follows:

(1) The ratio of compression is as follows:
  (8) $$\begin{array}{c} \text{RC}=\frac{M_{S}\times N_{S}}{f}=\frac{M_{S}\times N_{S}}{3\times M_{S}\times N_{S}}=\frac{1}{3}. \end{array}$$
(2) Since in the embedding phase, (*t* − 1) shared secret data embedded into one pixel of the cover image, thus *J* is (*t* − 1).
(3) The embedding capacity EC is shown as follows:
  (9) $$\begin{array}{c} \text{EC}=M_{C}\times N_{C}\times J\times \text{RC}=\frac{M_{C}\times N_{C}\times \left(t-1\right)}{3}. \end{array}$$

In the proposed scheme, the secret image is compressed through the use of the differential encoding, the Huffman coding, and the binary to hexadecimal data converting before sharing. The use of differential encoding transforms pixels of the secret image into the correlation between neighboring pixels of the secret image, which can improve the repetitive rate of secret data. The Huffman coding is applied to compress secret data that denotes the correlation. The higher the repetitive rate of secret data is, the better the compression effect is. Data converting is used to improve the performance of compression and adapts the proposed scheme to the power-of-two Galois Field GF(2^4^) well. Each 4 Huffman codes can be transformed into one 2^4^-ary notational system digit. Therefore, the ratio of compression RC depends on concrete compression operation and it is hard to precisely compute. However, through experiments shown later, the value of the ratio of compression RC is about 1.8 on average. And (*t* − 1)/2 shared secret data can be embedded into one pixel of the cover image. Therefore, we evaluate the embedding capacity EC of ours as follows:

(1) The ratio of compression is as follows:
  (10) $$\begin{array}{c} \text{RC}=\frac{M_{S}\times N_{S}}{f}\approx 1.8. \end{array}$$
(2) Since in the embedding phase, (*t* − 1) shared secret data embedded into one information datum and one information datum is obtained by two pixels of the cover image; thus *J* is (*t* − 1)/2.
(3) The embedding capacity EC is shown as follows:
  (11) ECMC×NC×J×RC≈MC×NC×t−1×1.82=0.9×MC×NC×t−1.

It is clearly that the embedding capacity of the proposed method is 0.9 × (*t* − 1) times of the size of the cover image, and it is bigger than that of Lin and Chan's scheme [14] which is (*t* − 1)/3 of the size of the cover image. A detailed comparison is shown in Table 3. Compared with Lin and Chan's scheme [14], the embedding capacity of the proposed scheme is improved largely because the secret image is compressed in the preprocessing phase before sharing it.

#### 4.1.2. Visual Quality

The visual quality of the stego images can be measured by the peak signal-to-noise (PSNR) [13, 14], which is shown in

(12) $$\begin{array}{c} \text{PSNR}=10\times \log_{10}\left(\;\frac{255^{2}}{\text{MSE}}\;\right), \end{array}$$

where the mean square error MSE is computed by

(13) $$\begin{array}{c} \text{MSE}=\frac{1}{M_{C}\times N_{C}}\overset{M_{C}-1\;}{\underset{i=0}{\sum }}\overset{\;N_{C}-1}{\underset{j=0}{\sum }}{\left(\;c_{i,j}-g_{i,j}\;\right)}^{2}. \end{array}$$

The higher the PSNR is, the better visual quality the stego image has. The PSNR is mainly determined by the difference range *L* = |*c*_*i*,*j*_ − *g*_*i*,*j*_|_max_, which can show the alteration of the pixel of the cover image after the embedding phase. The smaller the difference range *L* is, the higher PSNR is. The difference range *L* is determined by the embedding method.

In Lin and Chan's scheme [14], quantification embedding method is used. The difference range *L* is [−6,6] for gray scale covers. Binary covers cannot be used in their scheme. However, in the proposed scheme, the PMM embedding method is used and a new pixel mapping matrix is designed. The difference range is [−3,3] for both gray scale covers and binary covers.  Table 4 shows the difference range for gray scale covers and binary scale covers in detail.

From the above analysis, when using gray scale covers, it is clear that the PSNR of the proposed scheme is larger than that of Lin and Chan's scheme [14]. When using binary covers, the proposed scheme is also good, while Lin and Chan's scheme [14] is inapplicable. Thus, the visual quality of the proposed scheme is good for both gray scale covers and binary covers.

### 4.2. Experimental Results

**Figure 5:**
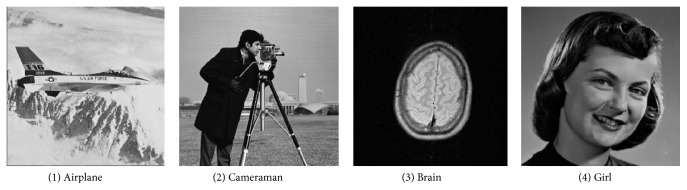
Secret images.

**Figure 6:**
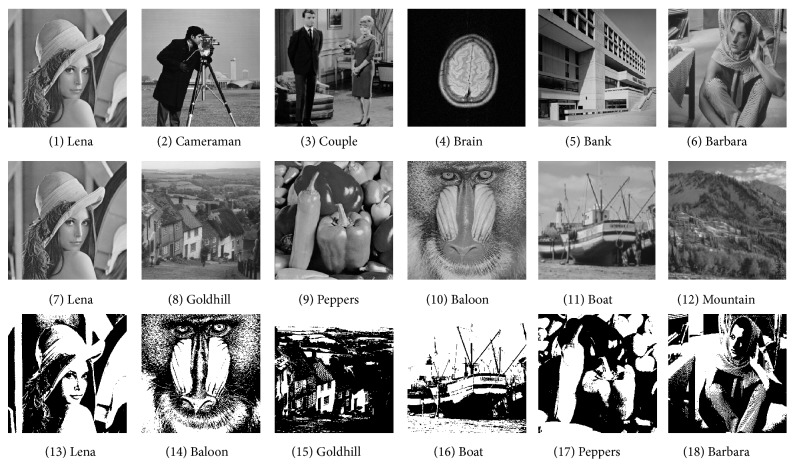
Cover images.

In this section, we shall use experiments to evaluate the performance of the proposed scheme, including the embedding capacity and the visual quality and then compare it with Lin and Chan's scheme [14]. Five experiments have been done. The first experiment in Section 4.2.1 is used to illustrate the embedding capacity of the proposed scheme and the second one in Section 4.2.2 is used to compare Lin and Chan's scheme [14] with the proposed scheme in embedding capacity, because Lin and Chan's scheme [14] has the largest embedding capacity among the existing schemes. The third experiment in Section 4.2.3 is used to illustrate the visual quality of the proposed scheme and the fourth one in Section 4.2.4 is used to compare Lin and Chan's scheme [14] with the proposed scheme in visual quality. The last one is shown in Section 4.2.3, and it displays the visual quality of the proposed scheme for binary covers that could not be used in Lin and Chan's scheme [14]. The detailed images used in five experiments are shown in Table 5 and Figures 5 and 6.

#### 4.2.1. Experiment on Embedding Capacity

**Figure 7:**
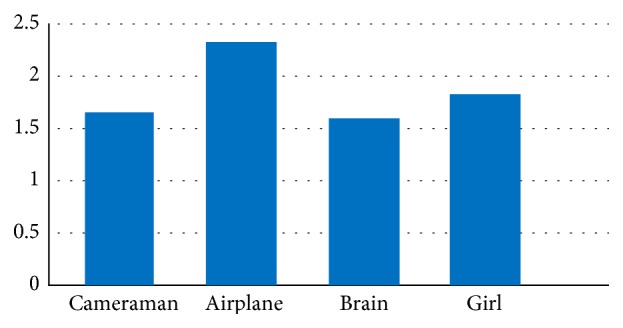
The value of RC.

Due to the discussion in Section 4.1.1, the embedding capacity is related to the ratio of compression RC. Therefore, in this experiment, we should firstly evaluate RC of the proposed scheme. Here, four different gray scale secret images shown in Figure 5 are used, respectively. For the proposed scheme, the value of the ratio of compression RC for different secret images is shown in Figure 7.

With the experiment results shown in Figure 7, the responding embedding capacity for different secret images can be got and shown in Table 6.

**Figure 8:**
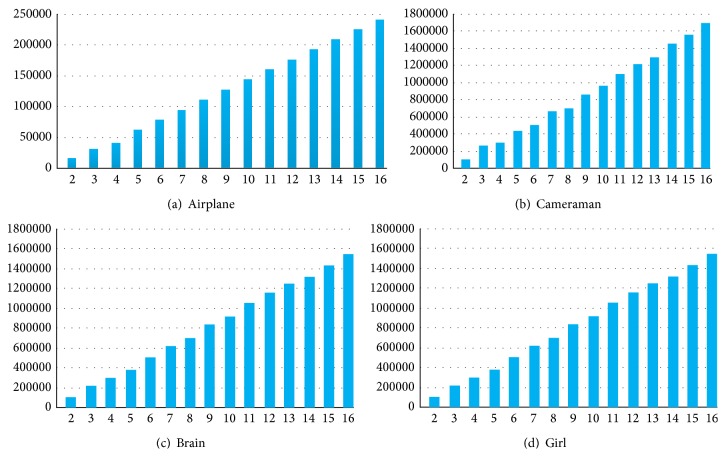
The embedding capacity EC with determined secret image.

From Figure 7, the largest value of RC can be 2.4 and the average value of RC can be 1.8. Table 6 shows that the largest embedding capacity can be 1.18 × (*t* − 1) of the size of the cover image in the proposed scheme. And the average embedding capacity is almost 0.9 × (*t* − 1) times of the cover image. Also, Figure 8 shows the embedding capacity with determined secret image for various values of *t*. It is clear that when the secret image and the cover image are determined, the embedding capacity EC rises with *t*.

#### 4.2.2. Comparison with Lin and Chan's Scheme in Embedding Capacity

**Figure 9:**
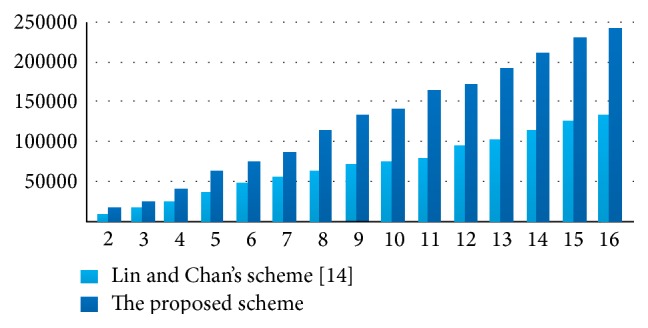
The comparison of the embedding capacity.

According to Section 2, we know that Lin and Chan's scheme [14] has the larger embedding capacity than the others of the existing schemes. So, in this experiment, we shall compare Lin and Chan's scheme [14] with the proposed scheme in embedding capacity. When using the same secret image and the same cover image, the comparison result of the embedding capacity of the proposed scheme with and Lin and Chan's scheme [14] for various *t* is shown in Figure 9, through which it is easy to see when *t* becomes larger, the proposed scheme offers a larger embedding capacity than Lin and Chan's scheme [14].

The embedding capacity of the proposed scheme is improved largely because the secret image is compressed in the preprocessing phase before it is shared. The difference between Lin and Chan's scheme [14] and our scheme lies in the compression phase. In Lin and Chan's scheme, they only used the BTS in the compression phase, while in our scheme the differencing function, the Huffman coding, and BTH are used to compress data. More operations make our scheme consume more time in embedding phase. We experiment on the images given in Section 4.2 and on our PC (CPU: Intel Pentium E5700 3 GHZ, RAM: 2 G). Through the experiment, the average embedding time of Lin and Chan's scheme is about 18.3 seconds, while that of our scheme is about 19.5 seconds. It is easy to find that our method is about 6.6% slower than Lin and Chan's scheme [14] in speed. But our scheme has the larger embedding capacity. From this point, the decrease in speed is reasonable and can be accepted.

#### 4.2.3. Experiment on Visual Quality

This experiment aims to show the good visual quality of the proposed scheme when the size of the secret image is the same as the cover image. Without loss of generality, this experiment adopts (3,4)-threshold scheme. We mainly use PSNR and SSIM to evaluate the visual quality of our scheme.  Table 7 shows the PSNR of stego images when using different covers which has the same size as the secret image.

**Figure 10:**
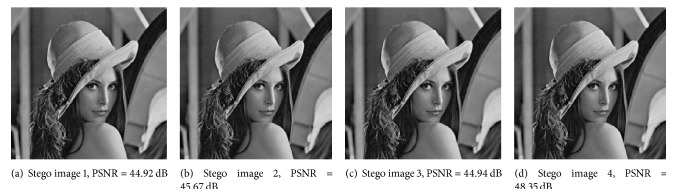
Stego images.

It can be seen that the PSNR of the stego image can be larger than 48 dB and the average value is almost 46 dB, which is much larger than the satisfying limitation, say 35 dB [14]. Therefore, the visual quality of the proposed scheme is good. More specifically, in order to show the visual effect, Figure 10 shows four generated stego images when using Figure 6(1) as the cover image.

**Figure 11:**
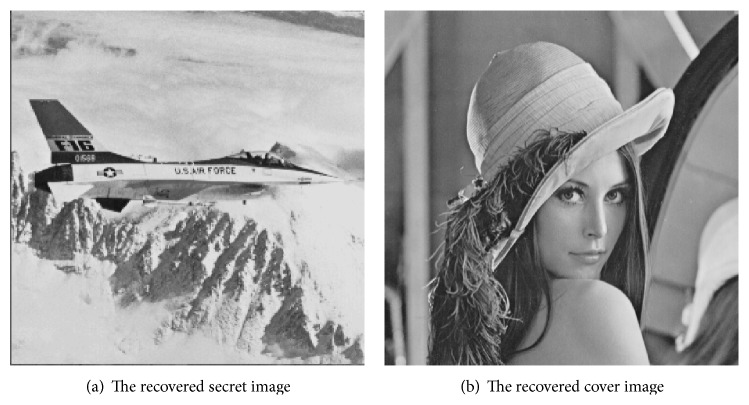
Recovered images.

From Figure 10, it is difficult for people to distinguish the cover image from stego images by eyes. Thus, the proposed scheme can cover up stego images from poachers. It means that the secret image could be effectively embedded into the cover image without easily attracting the attacker's attention.  Figure 11 shows the recovered secret and the recovered cover image when using any three of four stego images in Figure 10, which are shown in Figures 11(a) and 11(b), respectively. It can be seen that the secret image and the cover image are loss-less revealed.

At last, the evaluation results of the visual quality of the stego images by SSIM are given in Table 8.

From Table 8, it can be seen that SSIM of the stego images is 0.9506 at least and 0.9878 at most, which shows that the visual quality of the proposed scheme is good enough.

#### 4.2.4. Comparison with Lin and Chan's Scheme in Visual Quality

**Figure 12:**
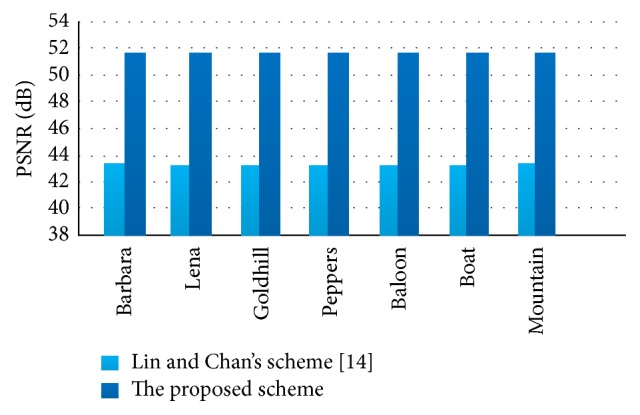
PSNR of two schemes for different grayscale cover images.

This experiment aims to display that the visual quality of the proposed scheme is better than Lin and Chan's scheme [14]. Also, without loss of generality, this experiment adopts (3, 4)-threshold scheme by using 7 different gray scale covers. The comparison of the visual quality of the proposed scheme with Lin and Chan's scheme [14] is shown in Figure 12.

From Figure 12, the proposed scheme generated stego images with approximately 51 dB PSNR, while Lin and Chan's scheme [14] generated stego images with 40 dB PSNR. It is clear that the proposed scheme is better than Lin and Chan's scheme [14] on the aspect of visual quality.

#### 4.2.5. Applications for Binary Covers

**Figure 13:**
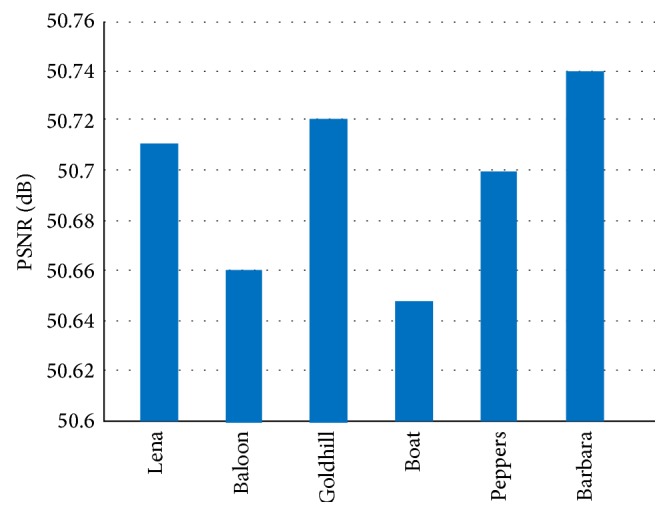
PSNR of the proposed scheme for different binary images.

This experiment aims to show that the visual quality of the proposed scheme is also good for binary covers that could not be used in Lin and Chan's scheme [14]. Without loss of generality, this experiment also adopts (3, 4)-threshold scheme with using 6 different binary covers. In Lin and Chan's scheme [14], the binary covers cannot be used due to underflow and overflow situations, while binary covers can be used in the proposed scheme. The PSNR of the proposed scheme for binary covers is shown in Figure 13.

From Figure 13, it can be seen that stego images in the proposed scheme could be formed with a high visual quality for binary covers.

In a word, through the performance analyses and experiments mentioned above, although there have been many schemes aiming to improve the embedding capacity with different methods by now, the proposed scheme offers the largest embedding capacity without underflow and overflow situations existing in Lin and Chan's scheme [14]. The comparison of the proposed scheme with the related schemes is shown in Table 9.

## 5. Conclusions

In this paper, we propose a new secret image sharing scheme to increase the embedding capacity and to eliminate the underflow and overflow situations at the same time. Firstly, the differential coding is applied to eliminate spatial redundancies of original secret data, and the Huffman coding is used to compress secret data. Meanwhile, data converting is used to adapt this scheme to the power-of-two Galois Field GF(2^4^) well. Through the above processes, the obtained data are smaller than the original one. At last, the PMM method is used to embed processed secret data into the cover image to avoid the underflow and overflow situations, where a new pixel mapping matrix is designed based on the improved exploiting modification direction (EMD) method and Sudoku method. Compared with the existing schemes, our scheme can improve the embedding capacity further and eliminate the underflow and overflow situations at the same time. Also, it can be used for binary covers.

## Figures and Tables

**Table 1:** The selected block.

	252	253	254	255
252	12	13	14	15
253	0	1	2	3
254	4	5	6	7
255	8	9	10	11

**Table 2:** The mapped block.

	252	253	254	255
252	12	13	14	15
253	0	1	2	3
254	4	5	6	7
255	8	9	10	11

**Table 3:** The comparison of the embedding capacity.

Schemes	Methods (before sharing)	*J*	RC	EC
Lin and Chan's scheme [14]	BTS	(*t* − 1)	1/3	*M*_*C*_ × *N*_*C*_ × (*t* − 1)/3

The proposed scheme	The differencing function The Huffman coding BTH	(*t* − 1)/2	1.8	0.9 × *M*_*C*_ × *N*_*C*_ × (*t* − 1)

**Table 4:** The difference range for gray scale covers and binary scale covers.

Schemes	Embedding method	Gray scale covers	Binary covers
Lin and Chan's scheme [14]	Quantification	[−6, 6]	—

The proposed scheme	PMM	[−3, 3]	[−3, 3]

**Table 5:** Used images.

Experiments	Secret image				Cover image			
	Name	Number	Kind	Size (pixels)	Name	Number	Kind	Size (pixels)
Exp. 1	Figure 5	4	Gray scale	256 × 256	Figure 6(1)	1	Gray scale	512 × 512
Exp. 2	Figure 5(1)	1	Gray scale	256 × 256	Figure 6(1)–(5)	5	Gray scale	256 × 256
Exp. 3	Figure 5(1)	1	Gray scale	256 × 256	Figure 6(1)	1	Gray scale	512 × 512
Exp. 4	Figure 5(1)	1	Gray scale	256 × 256	Figure 6(6)–(12)	7	Gray scale	512 × 512
Exp. 5	Figure 5(1)	1	Gray scale	256 × 256	Figure 6(13)–(18)	6	Binary	256 × 256

**Table 6:** The embedding capacity.

Secret image	Embedding capacity
Cameraman	0.7921 × (*t* − 1) × *M*_*C*_ × *N*_*C*_
Airplane	1.18 × (*t* − 1) × *M*_*C*_ × *N*_*C*_
Brain	0.7772 × (*t* − 1) × *M*_*C*_ × *N*_*C*_
Girl	0.8478 × (*t* − 1) × *M*_*C*_ × *N*_*C*_

**Table 7:** PSNR of stego images.

Cover image	The proposed scheme (dB)			
	Stego image 1	Stego image 2	Stego image 3	Stego image 4
Lena	44.92	45.67	44.94	48.35
Cameraman	44.93	45.68	44.98	48.39
Couple	45.07	45.76	45.47	48.49
Brain	44.71	45.56	44.74	48.13
Girl	44.83	45.67	44.91	48.31
Bank	44.87	45.64	44.93	48.31
Average	44.89	45.66	45.00	48.33

**Table 8:** SSIM of stego images.

Cover image	The proposed scheme			
	Stego image 1	Stego image 2	Stego image 3	Stego image 4
Lena	0.9668	0.9780	0.9689	0.9878
Cameraman	0.9516	0.9696	0.9523	0.9767
Couple	0.9518	0.9685	0.9528	0.9806
Brain	0.9507	0.9684	0.9534	0.9717
Girl	0.9549	0.9667	0.9656	0.9837
Bank	0.9506	0.9746	0.9678	0.9858

**Table 9:** Comparisons of the related schemes.

Method	Embedding capacity	Underflow and overflow situations
Lin and Tsai [4]	(1/4) × *M*_*C*_ × *N*_*C*_	no
Lin et al. [8]	((*t* − 3)/3) × *M*_*C*_ × *N*_*C*_	no
Lin and Chan [14]	((*t* − 1)/3) × *M*_*C*_ × *N*_*C*_	yes
Chang et al. [11]	((*t* − 1)/4) × *M*_*C*_ × *N*_*C*_	no
Guo et al. [10]	((*t* − 1)/4) × *M*_*C*_ × *N*_*C*_	no
Ulutas et al. [13]	(1/4) × *M*_*C*_ × *N*_*C*_	no
The proposed scheme	((*t* − 1) × RC/2) × *M*_*C*_ × *N*_*C*_	no

## Notations

*n*: The number of sharing participants
*t*: The threshold value
*P* = {*p*_0_*p*_1_ ⋯ *p*_*n*−1_}: The public identity of *n* participants, and in this paper, we set *p*_*i*_ = *i* + 1 for simplicity
*M*_*S*_: The length of the secret image *S*
*N*_*S*_: The width of the secret image *S*
*S* = {*s*_*i*,*j*_}: The secret image. Here, *s*_*i*,*j*_ ∈ {0,1,…, 255} for *i* = 0,1,…, *M*_*S*_ − 1, *j* = 0,1,…, *N*_*S*_ − 1
*D* = {*d*_*i*,*j*_}: The difference image, *i* = 0,1,…, *M*_*S*_ − 1, *j* = 0,1,…, *N*_*S*_ − 1, *d*_*i*,*j*_ ∈ {−255, −254,…, 255}
*B* = *b*_0_*b*_1_ … *b*_*M*_*S*_×*N*_*S*_−1_: The difference vector, *b*_*i*_ ∈ {−255, −254,…, 255}
*H* = *h*_0_*h*_1_ ⋯ *h*_len−1_: The Huffman code, *h*_*i*_ ∈ {0,1}
*R* = *r*_0_*r*_1_ ⋯ *r*_*u*−1_: The transformed secret data, *u* = ⌈len/4⌉, *r*_*i*_ ∈ {0,1,…, 15}
%: The modular arithmetic
*E* = *e*_0_*e*_1_ ⋯ *e*_*f*−1_: The shared secret data, *f* = *u* + (*t* − 1 − (*u* + 6)%(*t* − 1)) + 6, *e*_*i*_ ∈ {0,1,…, 15}
*Q* = *q*_0_*q*_1_ ⋯ *q*_*m*−1_: The information data, *m* = *f*/(*t* − 1), *q*_*i*_ ∈ {0,1,…, 15}
BTH(*x*): Transforming the binary number *x* into a hexadecimal number
HTB(*x*): Transforming a hexadecimal number *x* into the binary number
BTS(*x*): Transforming the binary number *x* into a Septenary number
mat_*i*,*j*_: The value in the pixel mapping matrix, *i* = 0,1,…, 255, *j* = 0,1,…, 255, mat_*i*,*j*_ ∈ {0,1,…, 15}
*M*_*C*_: The length of the cover image *C*
*N*_*C*_: The width of the cover image *C*
*C* = {*c*_*i*,*j*_}: The cover image, *i* = 0,1,…, *M*_*C*_ − 1, *j* = 0,1,…, *N*_*C*_ − 1, *c*_*i*,*j*_ ∈ {0,1,…, 255}
*Y* = *y*_0_*y*_1_ ⋯ *y*_*v*−1_: The embedded data, *v* = *f*/(*t* − 1) × *n*, *y*_*i*_ ∈ {0,1,…, 15}
*F*(*x*): Lagrange interpolation polynomial
*d*: The constant of the Lagrange interpolation polynomial
*a*_1_, *a*_2_,…, *a*_*t*−1_: The coefficient of the Lagrange interpolation polynomial
*G*^*i*^ = {*g*_*k*,*l*_^*i*^}: The *i*th stego image, *i* = 0,1,…, *n* − 1, *k* = 0, 1,…, *M*_*C*_ − 1, *l* = 0,1,…, *N*_*C*_ − 1, *g*_*k*,*l*_^*i*^ ∈ {0,1,…, 255}
*Z*^*i*^ = {*z*^*i*^}: The *i*th stego vector, *i* = 0,1,…, *n* − 1, *z* ^*i*^ ∈ {0,1,…, 255}
RC: The ratio of compression
EC: The embedding capacity
*J*: The number of shared secret data that can be embedded into one pixel of the cover image.

## References

[1] Blakley G. R. Safe guarding cryptographic keys.

[2] Shamir A. (1979). How to share a secret. *Communications of the ACM*.

[3] Naor M., Shamir A. (1995). Visual cryptography. *Advances in Cryptology—EUROCRYPT '94*.

[4] Lin C.-C., Tsai W.-H. (2004). Secret image sharing with steganography and authentication. *Journal of Systems and Software*.

[5] Wu Y.-S., Thien C.-C., Lin J.-C. (2004). Sharing and hiding secret images with size constraint. *Pattern Recognition*.

[6] Yang C.-N., Chen T.-S., Yu K. H., Wang C.-C. (2007). Improvements of image sharing with steganography and authentication. *Journal of Systems and Software*.

[7] Chang C.-C., Hsieh Y.-P., Lin C.-H. (2008). Sharing secrets in stego images with authentication. *Pattern Recognition*.

[8] Lin P.-Y., Lee J.-S., Chang C.-C. (2009). Distortion-free secret image sharing mechanism using modulus operator. *Pattern Recognition*.

[9] Eslami Z., Ahmadabadi J. Z. (2011). Secret image sharing with authentication-chaining and dynamic embedding. *Journal of Systems and Software*.

[10] Guo C., Wang Z.-H., Chang C.-C., Qin C. (2011). A secret image sharing scheme with high quality shadows based on exploiting modification direction. *Journal of Multimedia*.

[11] Chang C.-C., Lin P.-Y., Wang Z. H., Li M. C. (2010). A sudoku-based secret image sharing scheme with reversibility. *Journal of Communications*.

[12] Li L., Abd El-Latif A. A., Yan X., Wang S., Niu X. A lossless secret image sharing scheme based on steganography.

[13] Ulutas M., Ulutas G., Nabiyev V. V. (2013). Invertible secret image sharing for gray level and dithered cover images. *Journal of Systems and Software*.

[14] Lin P.-Y., Chan C.-S. (2010). Invertible secret image sharing with steganography. *Pattern Recognition Letters*.

[15] Lee C.-F., Wang Y.-R., Chang C.-C. A steganographic method with high embedding capacity by improving exploiting modification direction.

[16] Zhang X., Wang S. (2006). Efficient steganographic embedding by exploiting modification direction. *IEEE Communications Letters*.

[17] Mathematics of Sudoku http://en.wikipedia.org/wiki/Mathematics_of_Sudoku.

[18] Felgenhauer B., Jarvis F. (2006). Mathematics of sudoku I. *Mathematical Spectrum*.

[19] Russell E., Jarvis F. (2007). Mathematics of sudoku II. *Mathematical Spectrum*.

[20] Chang C.-C., Chou Y.-C., Kieu D. An information hiding scheme using Sudoku.

[21] Chen J., Chen T. S., Hsu H. C., Chen H. W. (2005). New visual cryptography system based on circular shadow image and fixed angle segmentation. *Journal of Electronic Imaging*.

[22] Wu H.-C., Chang C.-C. (2005). Sharing visual multi-secrets using circle shares. *Computer Standards & Interfaces*.

[23] Chen T.-H., Li K.-C. (2012). Multi-image encryption by circular random grids. *Information Sciences*.

[24] Liu F., Wu C. (2011). Embedded extended visual cryptography schemes. *IEEE Transactions on Information Forensics and Security*.

[25] Liu F., Wu C., Lin X. (2011). Cheating immune visual cryptography scheme. *IET Information Security*.

[26] Thien C.-C., Lin J.-C. (2002). Secret image sharing. *Computers & Graphics*.

[27] Wang R.-Z., Su C.-H. (2006). Secret image sharing with smaller shadow images. *Pattern Recognition Letters*.

[28] Chen C. C., Chen C. C., Lin Y. C. (2009). Weighted modulated secret image sharing method. *Journal of Electronic Imaging*.

[29] Shyu S. J., Chuang C. C., Chen Y. R., Lai A. F. (2009). Weighted threshold secret image sharing. *Advances in Image and Video Technology*.

[30] Lin S.-J., Chen L. S.-T., Lin J.-C. (2009). Fast-weighted secret image sharing. *Optical Engineering*.

[31] Li P., Yang C.-N., Wu C.-C., Kong Q., Ma Y. (2013). Essential secret image sharing scheme with different importance of shadows. *Journal of Visual Communication & Image Representation*.

[32] Chang C.-C., Lin C.-C., Lin C.-H., Chen Y.-H. (2008). A novel secret image sharing scheme in color images using small shadow images. *Information Sciences*.

[33] Guo C., Chang C.-C., Qin C. (2012). A hierarchical threshold secret image sharing. *Pattern Recognition Letters*.

[34] Pakniat N., Noroozi M., Eslami Z. (2014). Secret image sharing scheme with hierarchical threshold access structure. *Journal of Visual Communication & Image Representation*.

[35] Ulutas G., Ulutas M., Nabiyev V. V. (2013). Secret image sharing scheme with adaptive authentication strength. *Pattern Recognition Letters*.

[36] Chen G., Liu J., Wang L. (2012). Color image sharing method based on lagrange's interpolating polynomial. *Health Information Science*.

[37] Anbarasi L. J., Kannan S. Secured secret color image sharing with steganography.

[38] Kumar H., Srivastava A. A secret sharing scheme for secure transmission of color images.

[39] Wang Z., Bovik A. C., Sheikh H. R., Simoncelli E. P. (2004). Image quality assessment: from error visibility to structural similarity. *IEEE Transactions on Image Processing*.

[40] Chan C.-K., Cheng L. M. (2004). Hiding data in images by simple LSB substitution. *Pattern Recognition*.

[41] Chang C.-C., Hsiaob J.-Y., Chan C.-S. (2003). Finding optimal least-significant-bit substitution in image hiding by dynamic programming strategy. *Pattern Recognition*.

[42] Wang R.-Z., Lin C.-F., Lin J.-C. (2001). Image hiding by optimal LSB substitution and genetic algorithm. *Pattern Recognition*.

[43] Thien C.-C., Lin J.-C. (2003). A simple and high-hiding capacity method for hiding digit-by-digit data in images based on modulus function. *Pattern Recognition*.

[44] Chang C.-C., Chan C.-S., Fan Y.-H. (2006). Image hiding scheme with modulus function and dynamic programming strategy on partitioned pixels. *Pattern Recognition*.

